# Complete mitochondrial genome of *Eilema ussiricum* (Lepidoptera: Erebidae)

**DOI:** 10.1080/23802359.2020.1720546

**Published:** 2020-02-06

**Authors:** Xiao-Bin Zhang, Yu-Peng Wu

**Affiliations:** a Yuncheng University, Yuncheng, China;; b Shanxi Water Conservancy Technical Institute, Yuncheng, China

**Keywords:** *Eilema ussiricum*, complete mitogenome, Erebidae

## Abstract

The *Eilema ussiricum* belongs to Erebidae in Lepidoptera. The complete mitogenome of *E. ussiricum* was described in this study, which is typically circular duplex molecules and 15,344 bp in length, containing the standard metazoan set of 13 protein-coding genes, 22 transfer RNA genes, 2 ribosomal RNA genes, and an A + T-rich region. All the inferred tRNA secondary structures show the common cloverleaf pattern, with the exception of tRNA-Ser(AGN) which lacks the DHU arm. Except for *cox1* started with CGA, all other PCGs started with the standard ATN codons. All of the PCGs terminated with the stop codon TAA. The phylogenetic tree showed that the species of subfamily Arctiinae include *E. ussiricum* are clustered into a clade.

*Eilema ussuricum* is a moth of the family Erebidae. Erebidae was upgraded to family from Erebinae (Lafontaine and Fibiger 2006) within Noctuoidea and newly revised by some scholars (Zahiri et al. 2011). *Eilema ussuricum* is widely distributed in Russia, China, and Korea. However, its mitochondrial genome has not been reported publicly.

In this study, the mitochondrial genome of *E. ussuricum* was sequenced and annotated. The samples were collected in Taiyuan city of China (37°83′33″N, 112°66′61″E) in July 2019, some of these specimens were immediately frozen in −80 °C on board for mitogenome analysis and others were preserved by spreading wings in the Herbarium of Institute of Plant Protection, Shanxi Academy of Agricultural Sciences, and their numbers is 2019TYKD1701-1705. Total genomic DNA was extracted from tail tip using the Ezup pillar genomic DNA extraction kit (Sangon Biotech, Shanghai, China). The mitogenome was sequenced by Illumina Hiseq 4000. Gene annotation was performed and circularity was checked using the MITOS2 webserver (Bernt et al. 2013, http://mitos.bioinf.uni-leipzig.de/).

The mitochondrial genome of *E. ussiricum* has a total length of 15,344 bp (GenBank accesion No. MN696172), consisting of 13 PCGs, 22 tRNA, 2 rRNA genes, and an A + T-rich region. As with the other insect mitogenomes (Wu et al. 2016), the major strand encodes a larger number of genes (9 PCGs and 14 tRNAs) than the minor strand (4 PCGs, 8 tRNAs, and 2 rRNA genes). Two rRNAs (16S rRNA and 12S rRNA) are located between tRNA-Leu(CUN) and tRNA-Val and between tRNA-Val and the A + T-rich region, respectively. The 16S rRNA is 1308 bp in length and the 12S rRNA is 726 bp in length. The A + T-rich region is 412 bp long and located between 12S rRNA and tRNA-Met. The mitogenome contains 40.21% T, 40.25% A, 11.98% C, and 7.56% G. All of the protein**-**coding genes have ATN as the start codon except for *cox1*, which starts with CGA. All of the PCGs have the common stop codon TAA.

The phylogenetic position of *E. ussiricum* was inferred using sequences of the 13 PCGs of 15 species. Fourteen of them belong to Erebidae and a species *Spodoptera frugiperda* from Noctuidae (which was used as outgroup) (Figure 1). The sequences were aligned with MAFFT v7.2 software (Katoh and Standley 2013) and the evolutionary analyses were conducted with RAxML v8.2.10 (Stamatakis 2014) on the CIPRES Science Gateway (Miller et al. 2010). The result showed that the species of subfamily Arctiinae include *E. ussiricum* are clustered into a clade. Aganainae (represented by *Asota plana*) and Herminiinae (represented by *Hydrillodes lentalis*) are clustered into a clade. Hypeninae (represented by *Paragabara curvicornuta*) form another monophyly.

**Figure 1.**
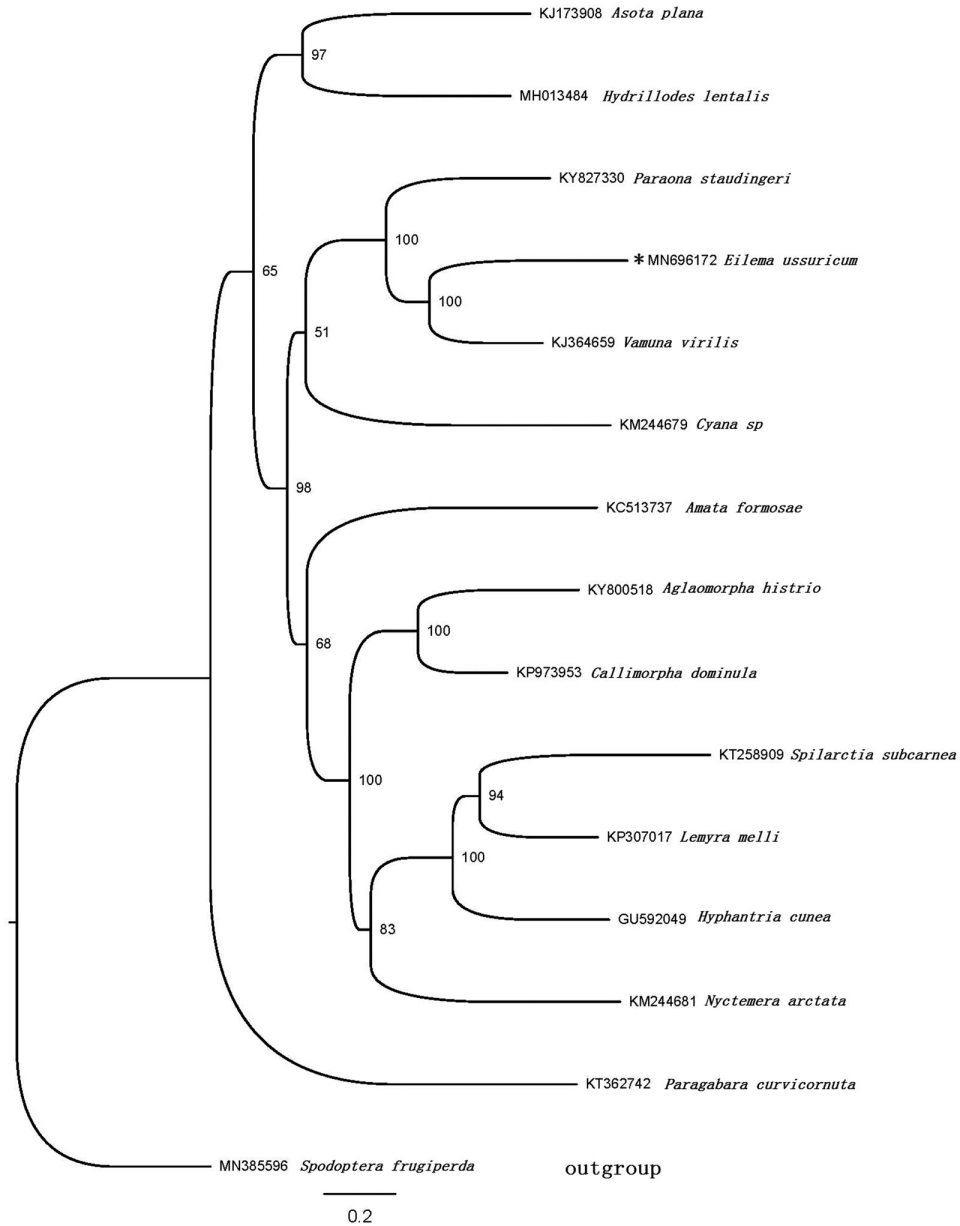
Maximum-likelihood tree of evolutionary relationships *E. ussiricum* based on the complete mitogenomes of 15 Lepidopteran moths.

## Nucleotide sequence accession number

The complete mitochondrial genome sequence of *E. ussiricum* was deposited in GenBank under the accession number MN696172.
